# Imaging tau pathology in Parkinsonisms

**DOI:** 10.1038/s41531-017-0023-3

**Published:** 2017-06-29

**Authors:** Sarah Coakeley, Antonio P. Strafella

**Affiliations:** 1 Research Imaging Centre, Campbell Family Mental Health Research Institute, Centre for Addiction and Mental Health, University of Toronto, Toronto, ON Canada; 2 Division of Brain, Imaging and Behaviour—Systems Neuroscience, Krembil Research Institute, UHN, University of Toronto, Toronto, ON Canada; 3 Morton and Gloria Shulman Movement Disorder Unit and E.J. Safra Parkinson Disease Program, Neurology Division, Dept. of Medicine, Toronto Western Hospital, UHN, University of Toronto, Toronto, ON Canada

## Abstract

The recent development of positron emission tomography radiotracers targeting pathological tau in vivo has led to numerous human trials. While investigations have primarily focused on the most common tauopathy, Alzheimer’s disease, it is imperative that testing also be performed in parkinsonian tauopathies, such as progressive supranuclear palsy, corticobasal degeneration, and frontotemporal dementia and parkinsonism linked to chromosome 17. Tau aggregates differ in isoforms and conformations across disorders, and as a result one radiotracer may not be appropriate for all tauopathies. In this review, we evaluate the preclinical and clinical reports of current tau radiotracers in parkinsonian disorders. These radiotracers include [^18^F]FDDNP, [^11^C]PBB3, [^18^F]THK-5317, [^18^F]THK-5351, and [^18^F]AV-1451 ([^18^F]T807). There are concerns of off-target binding with [^18^F]FDDNP and [^11^C]PBB3, which may increase the signal to noise ratio and thereby decrease the efficacy of these radiotracers. Testing in [^18^F]THK-5317, [^18^F]THK-5351, and [^18^F]AV-1451 has been performed in progressive supranuclear palsy, while [^18^F]THK-5317 and [^18^F]AV-1451 have also been tested in corticobasal degeneration patients. [^18^F]THK-5317 and [^18^F]THK-5351 have demonstrated binding in brain regions known to be afflicted with pathological tau; however, due to small sample sizes these studies should be replicated before concluding their appropriateness in parkinsonian tauopathies. [^18^F]AV-1451 has demonstrated mixed results in progressive supranuclear palsy patients and post-mortem analysis shows minimal to no binding to non-Alzheimer’s disease tauopathies brain slices.

## Introduction

There are many classes of tauopathies, all categorized by the accumulation of pathological tau in the brain. Differences in tau isoforms, tau pathology, and distributions of pathological tau within the central nervous system (CNS) account for the varying symptom manifestations across tauopathies; however, there is also frequent symptom overlap between many tauopathies. The most common tauopathy is Alzheimer’s disease (AD). Many other non-AD tauopathies are also classified as parkinsonian disorders such as progressive supranuclear palsy (PSP), corticobasal degeneration (CBD), and frontotemporal dementia (FTD) and parkinsonism linked to chromosome 17 (FTDP-17). Tauopathies such as PSP may have clinical signs that overlap with other parkinsonian disorders; therefore, a method of distinguishing between tauopathies and non-tauopathies would be very useful. Recently, there has been an increased interest in developing positron emission tomography (PET) radiotracers that bind tau in vivo. While many of these radiotracers have been developed and tested in AD, an increasing number of post-mortem and human studies are involving parkinsonian tauopathies. In this review we aim to examine the efficacy of tau radiotracers in parkinsonism and outline future directions for tau imaging.

## Tau and pathology

Tau is a microtubule-associated protein (MAP) found in the CNS and peripheral nervous system. It is primarily located in the axons of neurons in healthy conditions.^1^ Similarly to other MAPs, the role of tau is related to microtubule assembly and stabilization.^2, 3^ Alternative splicing of exon 10 produces isoforms with 3 amino acid repeats (3R) or 4 amino acid repeats (4R) on the carboxyl terminal. The repeats contain tubulin and microtubule binding domains; therefore, the 4R isoforms have a slightly higher affinity for microtubules than the 3R isoforms.^4, 5^ In a human brain free of tau pathology the ratio of 3R to 4R tau isoforms is approximately equal.^6^ Localization of tau in the neuron is largely dependent on post-translational modifications, most notably phosphorylation.^1^ Phosphorylation of tau decreases its affinity for microtubules, thereby decreasing overall tubulin assembly.^5^ Phosphorylated tau is generally sequestered in the soma of neurons, with small traces found in the nucleus—possibly involved in the regulation of *MAPT* mRNA transcription. Non-phosphorylated tau is found in distal axonal regions of neurons and is more prone to proteolysis than its phosphorylated counterpart.^1, 5^ Though the process of phosphorylation is reversible, hyperphosphorylation of tau can disrupt the structure of neuronal microtubules and lead to pathological conditions known as tauopathies.^1, 6^ Neurofibrillary tangles (NFT) are formed from phosphorylated tau aggregates and remain intracellular, until the death of the neuron.^1, 5^ In many tauopathies tau aggregates are not confined to gray matter but also present in glial cells, namely astrocytes and oligodendrocytes.

## Tau in Parkinsonism

### Progressive supranuclear palsy

PSP is the second most common parkinsonian disorder, following classic Parkinson’s disease (PD), and affects approximately 5–6/100,000 North Americans.^7, 8^ This neurodegenerative disorder often presents in middle to late age (55 to 70 years) and disease duration is approximately 4 to 5.5 years.^9^ Onset of PSP symptoms is insidious, the earliest sign of PSP is most commonly unexplained falls, followed by deteriorating postural stability. Other clinical manifestations include supranuclear gaze palsy, dysarthria, gait difficulty, and bradykinesia similar to PD.^10, 11^ Cognitive changes and eventually dementia can appear later on in the disease progression.^11, 12^ Misdiagnosis is a common problem in PSP and the most common misdiagnosis of PSP is PD.^9^ Although pathology at autopsy remains the gold standard for a diagnosis of PSP, the International Parkinson and Movement Disorder Society PSP Study Group recently released revised diagnostic criteria for PSP (MDS-PSP criteria).^11^ All diagnoses require a sporadic occurrence of the disease, with the first PSP-related symptom appearing after 40 years of age, a gradual progression of the disorder, and no evidence of clinical or imaging findings that could suggest another disorder (found under mandatory exclusion criteria). The MDS-PSP Study Group has identified 4 levels of diagnostic certainty: definite PSP, probable PSP, possible PSP, and suggestive PSP. A definite PSP diagnosis can only be obtained with neuropathological evidence. The other three levels of certainty require varying combinations of the following domains: ocular motor dysfunction, postural instability, akinesia, and cognitive dysfunction. Within each core clinical feature there are multiple levels of certainty outlined in the new criteria. A diagnosis of “probable PSP” is designed to be highly specific, and therefore is suitable for biological and therapeutic studies.^11^

Tau aggregates are the main pathology in PSP; therefore, it is classified as a primary tauopathy. In neurons, NFTs present as straight filaments and are formed primarily by 4R tau inclusions.^13–15^ Aggregation also occurs in glial cells—in astrocytes these are termed ‘tufted astrocytes’ and in oligodendrocytes ‘coiled bodies’. The regional distribution of lesions is not clustered and generally presents as random dispersions.^16, 17^ The majority of NFT tau pathology is situated in subcortical regions, primarily in the basal ganglia, with involvement specifically in the striatum, caudate, putamen, subthalamic nucleus, substantia nigra, and also dentate nucleus of the cerebellum.^16, 18, 19^

### Corticobasal degeneration

CBD is primarily a sporadic movement disorder that is difficult to diagnose clinically due to its lack of specificity and clinical heterogeneity.^15, 20^ Approximately 25–56% of cases are accurately diagnosed ante mortem.^20^ Cardinal signs of CBD include asymmetrical apraxia, rigidity, bradykinesia, and dystonia. Other clinical presentations consist of aphasia, alien limb phenomenon, cortical sensory deficit, focal myoclonus, and a lack of response to levodopa.^15, 20–23^ Cognitive impairment can develop during the disease progression, and may lead to a misdiagnosis of AD.^21, 22^ A probable CBS diagnosis requires two of the following symptoms with asymmetric presentation: limb rigidity or akinesia, limb dystonia, limb myoclonus; in addition to two of the following symptoms: orobuccal or limb apraxia, cortical sensory deficit, alien limb phenomena.^21^ Possible CBS criteria includes the same symptoms as above; however, only one symptoms from each group is required.^21^

Pathological diagnosis continues to be the gold standard for CBD, with tau aggregation being the primary source of pathology.^20, 24^ The main tau isoform present in CBD tau inclusions is 4R.^2, 3, 6, 8, 15, 20, 22, 25^ Straight filament NFTs, astrocytic plaques, and coiled bodies often accumulate in CBD in a clustered formation.^3, 16, 26^ Asymmetrical frontal and parietal lobe atrophy is seen in CBD cases.^23, 27^ Atrophy can also be seen in the thalamus, subthalamic nucleus, pallidum, dentate nucleus, and brainstem nuclei.^23^ The majority of CBD pathology is found in the gray and white matter of the cortex.^15, 28^

### FTD and parkinsonism linked to chromosome-17

FTD is the clinical term for a heterogeneous group of syndromes that present with non-Alzheimer’s dementia.^29^ Spillantini and colleagues (1998) described a new form of tauopathy labeled FTDP-17.^30^ Clinically this disorder presents with signs of FTD and parkinsonism without tremor. Although an initial response to levodopa may occur, patients often lose this response.^31^ FTDP-17 may exhibit clinical signs of PSP, CBD, and AD.^31^ FTDP-17 presents with tau aggregates as a result of a mutation in the *MAPT* gene on chromosome 17.^30, 31^

## Imaging tau pathology

PET is a non-invasive imaging modality that uses radioligands (radiotracers) to detect targets in vivo. Ligands are typically labeled with either fluorine-18 or carbon-11, radioisotopes with relatively short half-lives (110 and 20 min, respectively). Following a venous injection, the radiotracer enters the bloodstream and is transported to the brain (or any other target organ/region) where it interacts with its target protein.

### PET tau radiotracers

Brain PET radiotracers must meet many of the same requirements of drugs with targets in the brain to passively cross the blood–brain barrier (BBB) and be effective neuroimaging agents. Lipophilicity is one of the most important factors that determines whether a radioligand will successfully cross the BBB. A LogP of approximately 2.0–3.5 is optimal because the compound is lipophilic enough to rapidly cross the bilipid membrane, but not so lipophilic that the compound binds to plasma proteins, P-glycoprotein, or other nonspecific targets.^32–34^ Another condition for passively crossing the BBB is a low molecular weight (<500 Da).^32^ An optimal radiotracer will have rapid uptake and washout from the brain. For the majority of effective radioligands 5% of the injected dose is taken up into the brain 2 to 5 min post-injection.^33^ It is important that metabolism of a radiotracer occurs outside the brain and that the resulting metabolites are less lipophilic than the parent compound to avoid nonspecific binding in the brain.^32, 34^

Tau as a radioligand target poses many challenges. It is an intracellular protein; therefore, the radiotracer must not only be capable of passing through the BBB, but also neuronal and glial plasma membranes.^33, 34^ Due to the various concentrations of tau in different brain regions, an ideal radiotracer would have very high affinity for tau to limit the amount of radioactivity that must be injected into the subject. The tau radioligand should have a very high specificity for tau because it is often co-localized with other proteins that have β-sheet secondary structures.^33, 34^ Due to the involvement of pathological tau in glial cells, the radioligand should also have very low nonspecific binding in white matter.^34^ As mentioned, tau has numerous isoforms and may aggregate in different conformations and therefore, it may be very challenging for one tau radiotracer to successfully image all tauopathies.

### FDDNP

The radiotracer ^18^F-(2-(1-{6-{(2-[^18^F]fluoroethyl)(methyl)amino]-2-naphthyl}ethylidene)malononitrile) ([^18^F]FDDNP) was developed as a non-invasive biomarker to image NFTs and Aβ plaques in the brains of AD patients.^35^ An overview of tau studies using [^18^F]FDDNP can be found in Table 1. The first human testing of [^18^F]FDDNP occurred in 9 AD subjects and 16 neurologically healthy controls. This study demonstrated that the [^18^F]FDDNP parent compound crossed the BBB quite rapidly and appeared to be metabolized peripherally with no signs of metabolites crossing the BBB.^35^ Using autoradiographic methods, it was determined that [^18^F]FDDNP binding in vivo was consistent with immunohistochemical staining of NFTs and Aβ.^35^

**Table 1:** Main findings from PET tau studies using [^18^F]FDDNP

Paper	Tracer	Clinical population	Findings
Shoghi-Jadid (2002)^35^	[^18^F]FDDNP	AD (*n* = 9); HC (*n* = 16)	Crosses BBB, no metabolites cross BBB; Positive staining of NFTs and Aβ
Small (2006)^36^	[^18^F]FDDNP	AD (*n* = 25); MCI (*n* = 28); HC (*n* = 30)	↑ binding AD compare to MCI and HC; High uptake associated with tau pathology in AD
Kepe (2013)^37^	[^18^F]FDDNP	PSP (*n* = 15); PD (*n* = 9); HC (*n* = 5)	↑ binding in midbrain, subthalamic regions and cerebellar white matter in PSP

A later [^18^F]FDDNP study included AD patients (*n* = 25), mild cognitive impairment (MCI) patients (*n* = 28), and healthy control participants (*n* = 30).^36^ MCI patients exhibited significantly increased uptake of [^18^F]FDDNP compared to healthy controls, while uptake in AD patients was significantly higher than both MCI and healthy controls.^36^ Areas with particularly high uptake were regions known to have tangle and plaque involvement in AD. A neuropathological evaluation was performed on one participant who passed away 14 months following baseline testing. It was found that areas high in [^18^F]FDDNP binding were associated with high NFTs and plaque immunoreactivity.^36^

Kepe and colleagues (2013)^37^ reported a [^18^F]FDDNP study performed on parkinsonian patients (15 PSP, 9 PD, and 5 healthy age-matched controls).^37^ All participants were imaged for 65 min following a [^18^F]FDDNP injection. Results demonstrated significantly increased binding of [^18^F]FDDNP in the midbrain, subthalamic region, and cerebellar white matter in PSP patients. Additionally, patients with more severe PSP had greater [^18^F]FDDNP binding in cortical regions.^37^ These subcortical regions are consistent with PSP pathology and cortical involvement is indicative of PSP progression. The primary concern with imaging PSP and other parkinsonian disorders using [^18^F]FDDNP is its lack of specificity for tau over other proteins with β-sheet structures.

### PBB3

Phenyl/pyridinyl-butadienyl-benzothiazoles/benzothiazoliums (PBBs) are a group of compounds developed for tau labeling based on fluorescent screening for β-sheet binding capability.^38^ Reports using [^11^C]PBB3 to detect tau in vitro and in vivo have been summarized in Table 2. Testing in the brain stem of PS19 mice (4R isoform mutation) demonstrated the ability of PBBs to bind tau positive NFTs. Furthermore, fluorescent microscopy confirmed the high affinity of PBBs for tau aggregates.^38^ Additional optimization yielded [^11^C]PBB3 for PET use. Using PS19 mice and microPET [^11^C]PBB3 rapidly crossed the BBB and bound tau inclusions. Retention was significantly greater in PS19 mice compared to age-matched control mice.^38^ The first human testing of [^11^C]PBB3 was measured in comparison to [^11^C]Pittsburgh compound B (PIB) signal in AD and healthy controls. The standardized uptake value ratio (SUVR) retention patterns of [^11^C]PBB3 differed significantly from that of [^11^C]PIB, suggesting that [^11^C]PBB3 binds tau selectively over Aβ.^38^ Accumulation of [^11^C]PBB3 was seen in the medial and lateral temporal cortices, and the frontal cortex—consistent with the Braak staging theory of AD.^38, 39^ When tested in a corticobasal syndrome patient [^11^C]PBB3 retention was high in the neocortex and subcortical structures. The signal was greater in the right basal ganglia as compared to the left, which was consistent with the atrophy displayed on the patient’s MRI.^38^ [^11^C]PBB3 must be tested in more patients with CBD and other parkinsonian disorders in order to validate its use in non-AD tauopathies. However, in a human trial with arterial blood sampling the parent compound was found to give rise to a more lipophilic metabolite known to cross the BBB in mouse brains.^40^ As previously mentioned, entry of a metabolite into the brain decreases the signal to noise ratio. Other radiotracers with optimal metabolites may be more effective at imaging tau pathology in parkinsonism.

**Table 2:** Main findings from PET tau studies using [^11^C]PBB3

Paper	Tracer	Clinical population	Findings
Maruyama (2013)^38^	[^11^C]PBB3	AD (*n* = 3); HC (*n* = 3)	SUVR pattern differed significantly from [^11^C]PIB; Binding consistent with tau pathology in AD;
		CBD (*n* = 1)	↑ binding in neocortex and subcortical structures
Kimura (2015)^40^	[^11^C]PBB3	Mice brains	Metabolite crosses BBB
Ono (2017)^71^	[^11^C]PBB3	In vitro	↑ binding of PBB3 compared to AV-1451; ↑ favorable binding to PSP tau sites to PBB3 compared to AV-1451

### THK derivatives

A group of tau radiotracers have been developed from arylquinone derivatives. The main findings from tau studies using THK derivatives are presented in Table 3. The first in its group to be tested was [^18^F]THK-523. Favorable properties of [^18^F]THK-523 as a radiotracer include low molecular weight, capability of being labeled with ^18^F at a high specific radioactivity, and an acceptable lipophilicity to cross the BBB.^41^ In vitro binding studies using AD and age-matched control brain slices and in vivo transgenic mice studies demonstrated that [^18^F]THK-523 selectively bound tau deposits over Aβ plaques.^41, 42^ Human testing in older adults (*n* = 10), semantic dementia (*n* = 3), and AD (*n* = 3) demonstrated elevated uptake of [^18^F]THK-523 in regions known to be afflicted with tau pathology in AD.^43^ However, testing in non-AD tauopathy brain slices (Pick’s disease (PiD), PSP, and CBD) revealed no THK-523 fluorescence.^44^ Lack of binding in these straight filament tauopathies may be due to a specificity of THK-523 for paired helical filament (PHF) tau conformation.

**Table 3:** Main findings from PET studies tau using THK derivatives

Paper	Tracer	Clinical population	Findings
Fodero-Tavoletti (2011)^41^	[^18^F]THK-523	In vitro AD (*n* = 3), HC (*n* = 3)	Crosses BBB; Selectivity for tau over Aβ
Harada (2013)^42^	[^18^F]THK-523	Transgenic mice	Selectivity for tau over Aβ
Villemagne (2014)^43^	[^18^F]THK-523	AD (*n* = 3), semantic dementia (*n* = 3), HC (*n* = 10)	↑ binding consistent with AD pathology
Fodero-Tavoletti (2014)^44^	[^18^F]THK-523	In vitro CBD (*n* = 2), PSP (*n* = 1), Pick’s disease (*n* = 1), AD (*n* = 3), PD (*n* = 2)	No THK-523 fluorescence in non-AD tauopathies
Okamura (2013)^45^	[^18^F]THK-5105, [^18^F]THK-5117	In vitro AD (*n* = 2), HC (*n* = 1)	Labeling NFTs in hippocampus of AD; Binding pattern differed significantly from [^11^C]PIB
Harada (2015)^46^	[^18^F]THK-5117	AD (*n* = 8), HC (*n* = 6)	SUVR pattern differed significantly from [^11^C]PIB; ↑ binding in temporal cortex of AD compared to HC; Off target binding in white matter
Ishiki (2015)^47^	[^18^F]THK-5117	AD (*n* = 5)	↑ SUVR in temporal cortex of AD compared to HC; Binding consistent with AD tau pathology; ↑ annual SUVR from medial to lateral temporal cortex
Saint-Aubert (2016)^48^	[^18^F]THK-5317	AD (*n* = 9), amnestic MCI (*n* = 11)	Binding consistent with AD tau pathology
Chiotis (2016)^49^	[^18^F]THK-5317	CBD (*n* = 1), PSP (*n* = 1), AD (*n* = 9), MCI (*n* = 13), young HC (*n* = 5), old HC (*n* = 4)	Minimal within-individual variability (test-retest); Binding consistent with CBD and PSP pathology
Harada (2015)^50^	[^18^F]THK-5351	AD (*n* = 3), HC (*n* = 3)	Autoradiography signal matching tau tangles; ↑ binding in temporal lobe in AD compared to HC
Ishiki (2016)^51^	[^18^F]THK-5351	PSP (*n* = 3), AD (*n* = 13), HC (*n* = 9)	Binding consistent with tau pathology in PSP; [^3^H]THK-5351 binding consistent with PSP tau IHC

Four derivatives of THK-523: THK-5105, THK-5117, THK-5317, and THK-5351 were optimized for PET use. Fluorescent tissue staining and autoradiography revealed that THK-5105 and THK-5117 labeling of NFTs in the hippocampus of AD brain slices was consistent with immunohistochemistry (IHC) results. There were significantly different binding patterns of [^18^F]THK-5105 and [^18^F]THK-5117 compared to [^11^C]PIB, suggesting that the arylquinone derivatives do not exhibit Aβ plaque binding.^45^ In a clinical study [^18^F]THK-5117 retention differed from that of [^11^C]PIB and the [^18^F]THK-5117 SUVRs of the temporal cortex were significantly higher in AD patients compared to healthy controls.^46^ High pathological tau load in the temporal cortex is associated with cognitive decline. [^18^F]THK-5117 demonstrated excellent pharmacokinetics, with rapid entry into the brain and reaching a plateau in healthy controls around 50 min post-injection. However, there was substantial retention in the subcortical white matter, which may represent off-target binding.^46^ A longitudinal [^18^F]THK-5117 study was performed by Ishiki and colleagues (2015) to track pathological tau in 5 cases of AD.^47^ Baseline scans revealed significantly higher SUVRs in the temporal lobes of AD patients compared to healthy controls. Additionally, regional distribution of [^18^F]THK-5117 in AD patients was consistent with previous post-mortem findings. Annual changes in the retention of [^18^F]THK-5117 in AD brains were seen in the middle and inferior temporal gyri and the fusiform gyrus, suggesting that tau pathology originates in the medial temporal cortex and accumulates laterally.^47^ Reports of [^18^F]THK-5117 in parkinsonian disorders have not been published; however, due to the possible off-target binding in white matter, this radiotracer may not be optimal for imaging disorders such as PSP and CBD, which present with white matter tau pathology.

Another analog of the THK family of radiotracers, [^18^F]THK-5317, has been developed and tested in humans. In one study involving AD and amnestic MCI patients, participants underwent [^18^F]THK-5317, [^18^F]FDG, and [^11^C]PIB scans.^48^ [^18^F]THK-5317 uptake was elevated in areas consistent with the Braak staging theory and was found to be significantly associated with cognition.^39, 48^ Another human study recruited patients with AD and MCI, one patient with a clinical diagnosis of CBD and another with PSP, as well as old and young healthy controls.^49^ The patient with CBD presented with signs of atypical parkinsonism predominantly on the left side, progressive apraxia and executive deficits. The PSP patient presented with progressive postural instability and falls, vertical supranuclear opthalmoaresis, dysdiadokinesia, and executive deficits.^49^ Distribution volume ratios (DVR) from 30–60 min post-injection were used to quantify uptake of [^18^F]THK-5317. Test-retest demonstrated minimal within-individual variability in [^18^F]THK-5317 DVR values. In the CBD patients, [^18^F]THK-5317 retention was seen in the basal ganglia, white matter of the cerebellum and other white matter areas. In the PSP patient retention was highest in the basal ganglia and the midbrain.^49^ While retention of [^18^F]THK-5317 reflects the known tau pathology of CBD and PSP, further investigation must be performed with a larger sample size to determine its utility in these disorders and establish any correlations with clinical measures, such as motor scores and cognition.

In a preclinical study [^18^F]THK-5351 demonstrated favorable pharmacokinetics and [^3^H]THK-5351 correlated with tau immunochemistry.^50^ [^18^F]THK-5351 demonstrated higher cortical-to-white matter binding compared to [^18^F]THK-5117, and although peak brain uptake was higher with [^18^F]THK-5117, [^18^F]THK-5351 demonstrated faster washout.^50^ Clinical testing in 3 AD and 3 healthy control subjects revealed binding of [^18^F]THK-5351 in brain regions associated with tau pathology in AD.^50^ [^18^F]THK-5351 was tested in a human study involving PSP patients (*n* = 3), AD patients (*n* = 13), and healthy controls (*n* = 9).^51^ All PSP participants presented with supranuclear gaze palsy and gait instability. There was significant retention of [^18^F]THK-5351in the globus pallidus and the midbrain of PSP patients.^51^ Additional autoradiography on separate PSP brain slices revealed that [^3^H]THK-5351 signal in the midbrain was highly correlated with IHC staining of tau aggregates.^51^ Continued investigation into the efficacy of [^18^F]THK-5351 imaging in PSP and other Parkinsonian tauopathies should involve increased sample size and correlations between ante-mortem and post-mortem data.

### [^18^F]AV-1451 ([^18^F]T807)

In attempt to develop a PET radiotracer designed to bind PHF tau, benzo[4,5]imidazole[1,2-a]pyrimidines fluorescent compounds were screened against PHF tau in AD brains yielded T726, which demonstrated selective binding to tau over Aβ plaques. Further optimization of T726 produced the non-fluorescent analog T807 (AV-1451).^52^ A summary of [^18^F]AV-1451 tau studies is presented in Table 4. Autoradiography revealed signals only seen in the PHF-tau rich slices, indicating a >25 fold selective binding of [^18^F]-T807 to tau over Aβ plaques.^52^ Pharmacokinetic evaluation revealed promising properties to cross the BBB.^52^ Overall preclinical evaluation of this radiotracer yielded promising results that [^18^F]T807 would effectively bind PHF-tau in human brains.

**Table 4:** Main findings from PET tau studies using [^18^F]AV-1451

Paper	Tracer	Clinical population	Findings
Xia (2013)^52^	[^18^F]AV-1451	In vitro	Autoradiography signal matching PHF tau; Favorable pharmacokinetics
Marquie (2015)^53^	[^18^F]AV-1451	In vitro CBD (*n* = 2), PSP (*n* = 3), AD (*n* = 3), HC (*n* = 2)	Autoradiography consistent with tau pathology in AD; No signal in non-AD (non PHF-tau) taupopathies; Binding to neuromelanin-containing cells in all cases
Sander (2016)^54^	[^18^F]AV-1451	In vitro CBD (*n* = 4), PSP (*n* = 6), Pick’s disease (*n* = 5), AD (*n* = 5)	Autoradiography signal consistent with tau pathology in AD and Pick’s disease; No signal in CBD and PSP
Lowe (2016)^55^	[^18^F]AV-1451	In vitro CBD (*n* = 2), FTLD-17 (*n* = 5), PSP (*n* = 3), AD (*n* = 3), HC (*n* = 3)	Signals consistent with tau pathology in AD and aging; No signal in non-AD tauopathies; Binding in midbrain in all cases
Chien (2013)^56^	[^18^F]AV-1451	AD (*n* = 2), MCI (*n* = 1), HC (*n* = 3)	Binding consistent with AD tau pathology; ↑ SUVR in temporal lobes and hippocampal area in AD compared to MCI and HC
Schwarz (2016)^57^	[^18^F]AV-1451	AD (*n* = 44), MCI (*n* = 87), old HC (*n* = 42)	Binding consistent with tau pathology in AD and HC aging
Sepulcre (2016)^58^	[^18^F]AV-1451	Old HC (*n* = 88)	Binding associated with atrophy in temporal lobe
Scholl (2016)^59^	[^18^F]AV-1451	Young HC (*n* = 5), old HC (*n* = 33)	↑ binding in medial temporal lobe related to aging; Binding associated with cognitive decline
Cho (2016)^60^	[^18^F]AV-1451	AD (*n* = 20), MCI (*n* = 15), HC (*n* = 20)	Binding consistent with tau pathology in AD and MCI; ↑ binding in AD compared to MCI and HC; ↑ binding in MCI compared to HC; Binding associated with cognitive decline and cortical atrophy
Ossenkoppele (2016)^61^	[^18^F]AV-1451	AD/MCI subtypes (*n* = 20), HC (*n* = 15)	Binding varied between AD/MCI subtypes; Binding in medial temporal lobe associated with aging
Chhatwal (2016)^62^	[^18^F]AV-1451	Old HC (*n* = 31)	Binding correlated with cerebrospinal fluid total-tau and phosphorylated-tau in temporal lobe
Smith (2016)^63^	[^18^F]AV-1451	PSP (*n* = 11), HC (*n* = 11)	↑ SUVR in globus pallidus, putamen, thalamus and midbrain in PSP compared to HC; Binding consistent with tau pathology in PSP and correlated with PSPRS scores; Overlap in binding between PSP and HC
Whitwell (2016)^64^	[^18^F]AV-1451	PSP (*n* = 10), AD (*n* = 10), HC (*n* = 50)	Binding consistent with tau pathology in PSP and correlated with PSPRS scores; ↑ binding in PSP compared to HC; Overlap in subcortical binding between PSP and HC; PSP SUVR low relative to high binding in AD
Cho (2016)^65^	[^18^F]AV-1451	PSP (*n* = 15), PD (*n* = 15), HC (*n* = 15)	Binding consistent with tau pathology in PSP; ↑ binding in PSP compared to PD and HC; No correlations with PSPRS scores
Coakeley (2016)^66^	[^18^F]AV-1451	PSP (*n* = 6), PD (*n* = 6), HC (*n* = 10)	No significant differences in PSP binding compared to PD and HC; No correlations with PSPRS scores
Passamonti (2017)^67^	[^18^F]AV-1451	PSP (*n* = 19), AD (*n* = 9), MCI (*n* = 6)	↑ binding in cortical regions of AD and MCI compared to PSP; ↑ binding in midbrain, putamen, pallidum, thalamus, dentate nucleus in PSP compared to AD and MCI; No correlations with PSPRS scores
Marquié (2017)^68^	[^18^F]AV-1451	PSP (*n* = 2), *MAPT* carrier (*n* = 1)	↑ binding in substantia and midbrain in PSP and *MAPT* carrier; No autoradiography signal in PSP and *MAPT* carrier
Josephs (2016)^69^	[^18^F]AV-1451	CBD (*n* = 1)	Binding consistent with tau pathology in CBD; Binding correlated with tau pathology at autopsy; Autoradiography signal in 3R tau isoform but not 4R tau isoform
McMillan (2016)^70^	[^18^F]AV-1451	CBD (*n* = 1)	Binding consistent with tau pathology in CBD; ↑ binding in follow-ups compared to baseline scan; Binding correlated with tau pathology at autopsy
Ono (2017)^71^	[^18^F]AV-1451	In vitro PSP, CBD, Pick’s disease, FTDP-17 with N279 and G272 *MAPT* mutation	↑ signal of PBB3 in non-AD tauopathies compared to AV-1451 signal
Vermeiren (2015)^72^	[^18^F]AV-1451	In vitro	Possible off-target binding to MAO-A

A post-mortem study by Marquie and colleagues (2015) performed further autoradiography screenings and included brain slices from an array of tauopathies. Brain slices from 3 AD, 3 PiD, 3 PSP, 2 CBD, and 2 control cases were tested. A phosphor screen [^18^F]AV-1451 autoradiography indicated strong signals in the entorhinal, frontal, temporal, parietal, and occipital cortices of AD brain slices with tau positive NFTs. Non-radiolabeled AV-1451 was able to competitively inhibited the [^18^F]AV-1451 signals.^53^ As expected, there was no signal in the control brain slices, with the exception of the substantia nigra, which was confirmed to be neuromelanin containing-cells. Non-PHF tauopathies (PiD, PSP, CBD, and transgenic mouse model) exhibited no signal of [^18^F]AV-1451 in brain regions afflicted with their respective tau pathology. However, in the PSP cases there was a signal in the entorhinal cortex, suggesting that [^18^F]AV-1451 was labeling age-related tau accumulation according the Braak staging theory.^39, 53^ It was concluded from this preclinical study that [^18^F]AV-1451 bound with selectivity to PHF-tau in AD and age-related tau accumulation, but did not bind to any significant degree to straight filaments of tau that are present in non-AD tauopathies (PiD, PSP, and CBD), and aggregates containing β-amyloid and α-synuclein. Additional binding to neuromelanin-containing cells, such as those in the substantia nigra pars compacta was observed.^53^ Another post-mortem examination of [^18^F]-AV-1451 in AD (*n* = 5), PSP (*n* = 6), PiD (*n* = 5), and CBD (*n* = 4) was performed by Sander and colleagues (2016).^54^ [^18^F]AV-1451 signal was consistent with IHC staining of tau in AD and PiD slices. However, there was no substantial signal in PSP and CBD brain slices, consistent with the previous post-mortem study.^54^ Lowe and colleagues (2016) tested AV-1451 binding in 38 cases, including age-related tauopathies, AD, PiD, CBD, PSP, FTDP-17, frontotemporal lobar degeneration with TDP-43 immunoreactive lesions (FTLD-TDP), lewy body disease (LBD), and multiple system atrophy (MSA).^55^ Cases with age-related tauopathies and AD cases demonstrated binding of AV-1451 to areas of high PHF concentration. Little to no specific binding was seen in PiD, CBD, and PSP. In some cases very minimal binding was seen in areas with PHF tangles. In FTDP-17 cases, binding was seen only in areas with PHF immunoreactivity. There was minimal AV-1451 signal in the frontal, parahippocampal and temporal cortices of the FTLD-TDP cases. The synucleinopathy cases (LBD and MSA) did not show any AV-1451 signal in brain areas with high α-synuclein immunoreactivity. There was binding in the midbrain of all cases, indicating the potential off target binding of AV-1451 to neuromelanin-containing cells.^55^

[^18^F]T807 was first tested clinically in 2 AD, 1 MCI, and three healthy controls.^56^ All participants underwent a dynamic PET scan from 0–60 min following the injection of [^18^F]T807 and a subsequent 80–100 min static scan was also acquired. In the MCI patient SUVR was increased in the hippocampal area, and the parietal, mesial temporal, and lateral temporal lobes. The mild AD patient had the highest SUVR in the lateral temporal lobe, and additionally had high SUVRs in the mesial temporal, parietal, frontal, and occipital lobes, and the hippocampal area. The volume of interest (VOI) with the most elevated SUVRs in the severe AD patient were the parietal and lateral temporal lobes, followed by the mesial temporal, frontal, and occipital lobes, and the hippocampal area.^56^ The severe AD patient had a significantly higher SUVR in the parietal lobe compared to the mild AD and the MCI patients. The pattern of [^18^F]T807 uptake in the severe AD patient reflected the pathological tau deposition outline in Braak stages V-VI.^39, 56^ The mild AD patient had significantly higher SUVR in the lateral temporal, mesial temporal lobes, and the hippocampal area compared to the MCI patient and healthy controls. This retention distribution is characterized by Braak stages III-IV.^39, 56^ Interestingly, the oldest healthy control (67 years old) had a higher SUV in the cerebellum, as well as the cortical VOIs compared to the two younger healthy controls (56 and 58 years old). This is thought to be due an age-related accumulation of PHF tau.^56^ Another human study was performed with AD patients (44), MCI patients (87) and healthy older adults (42) to test whether [^18^F]AV-1451 binding reflected the Braak histopathological staging.^39, 57^ The older control group was defined as individuals ≥50 years old. Distribution of [^18^F]AV-1451 SUVR (80–100 min) in all groups was in agreement with Braak staging patterns and previous neuropathological studies. Of all the subjects, only 7 presented with signals that did not conform to the neuropathological staging of tau deposition.^57^ [^18^F]AV-1451 has since been used to measure neurodegeneration and patterns of tau pathology in AD, MCI, and older healthy controls compared to younger healthy controls.^58–62^

[^18^F]AV-1451 has been tested in PSP patients in six separate studies. One study involving 11 PSP and 11 healthy age-matched controls found significantly higher retention of [^18^F]AV-1451 in the globus pallidus, putamen, thalamus, and midbrain of PSP patients and retention in the globus pallidus was found to be positively correlated with PSP Rating Scale (PSPRS) scores.^63^ However, there was found to be overlap in retention between PSP and healthy controls and no significant cortical retention, even in severe cases of PSP. Furthermore, [^3^H]AV-1451 autoradiography on 3 PSP brains revealed no specific binding.^63^ Similar results were also presented in a study with 10 probable PSP patients, 10 AD patients, and 50 healthy controls.^64^ PSP patients presented with higher retention in the midbrain, pallidum, thalamus, supplementary motor area, dentate nucleus of the cerebellum, precentral cortex, frontal inferior opercularis, caudate nucleus, and middle frontal gyrus when compared to healthy controls and uptake in these regions also correlated with PSPRS scores. However, again there was overlap between the PSP and healthy controls due to the subcortical areas of healthy controls presenting with age-related tau pathology.^64^ When compared to AD patients, PSP patients demonstrated increased retention in the dentate nucleus of the cerebellum and the pallidum. The SUVR of PSP patients was low relative to the high binding seen in AD patients.^64^ A study involving 15 PSP and 15 PD patients and 15 age-matched healthy controls reported increased binding in the putamen, globus pallidus, subthalamic nucleus, and dentate nucleus in PSP compared to both PD and healthy controls.^65^ PSP patients also had increased uptake in the substantia nigra compared to PD patients, which was interpreted to be off-target binding to neuromelanin. There were no significant increases in any cortical regions of the PSP group and no correlations with PSPRS scores or measures of cognition.^65^ Another clinical study with PSP (*n* = 6) patients, PD (*n* = 6) patients, and healthy controls (*n* = 10) reported no significant differences in [^18^F]AV-1451 retention in PSP patients compared to PD or healthy controls.^66^ There were also no correlations between [^18^F]AV-1451 retention and PSPRS or cognition scales.^66^ A recent study compared the binding of [^18^F]AV-1451 in AD (*n* = 9), MCI (*n* = 6), and PSP (*n* = 19).^67^ AD and MCI groups demonstrated increased binding potential (BP_ND_) in cortical regions compared to PSP, while greater binding was seen in the midbrain of the PSP group compared to AD. PSP also demonstrated higher binding in the putamen, pallidum, thalamus, midbrain, and dentate nucleus compared to healthy controls.^67^ However, no correlation between binding in PSP and UPDRS was found. Autoradiography was consistent with previous studies and revealed no specific binding in PSP.^67^ The sixth clinical study performed with [^18^F]AV-1451 in PSP patients provided both in vivo and post-mortem data on 2 PSP patients and one *MAPT* P301L mutation carrier to elucidate whether [^18^F]AV-1451 is able to bind tau in non-AD tauopathies (non-PHF).^68^ Both PSP patients demonstrated marked increased binding in the basal ganglia and midbrain, with lower signal in the frontal and temporal cortices, and the dentate nuclei.^68^ The *MAPT* mutation carrier also showed a similar pattern of uptake; however, all three subjects had lower retention in comparison to AD patients. Post-mortem autoradiography revealed no signal in either of the PSP patients and the *MAPT* mutation carrier, with the exception of binding to age-related tau pathology (signal in the entorhinal cortex), and the substantia nigra (off-target binding to neuromelanin).^68^

Currently there are only two case reports testing [^18^F]AV-1451 in CBD. One case study involved a CBD patient presenting with primary progressive apraxia of speech, difficulty swallowing, ideomotor apraxia, marked parkinsonism, and problems with balance and gait. This patient was scanned with [^18^F]AV-1451 14 months prior to death.^69^ Elevated [^18^F]AV-1451 SUVR was seen in the putamen, pallidum, thalamus, precentral cortex, rolandic operculum, supplemental motor area, and left Broca’s area. There were significant correlations between these regions of interest and tau burden evaluated at autopsy. However, autoradiographic testing indicated that [^18^F]AV-1451 binds predominantly to 3R tau isoforms, with minimal binding to the 4R isoform.^69^ Another case study performed by McMillan and colleagues (2016) presented similar results.^70^ The patient was scanned with [^18^F]AV-1451 15 months and 5 months prior to death and IHC was performed post-mortem. The first scan presented with high retention in the bilateral substantia nigra, globus pallidus and midbrain, while the 5 month scan demonstrated increased binding in cortical regions and the midbrain and pons.^70^ Post-mortem analysis confirmed the diagnosis of CBD and regions of high SUVR correlated significantly with the IHC results.^70^

In light of the mixed results of [^18^F]AV-1451 binding to non-AD tauopathies, an in vitro study compared the binding patterns of [^11^C]PBB3 and [^18^F]AV-1451.^71^ PBB3 demonstrated increased labeling of 4R tauopathies (PSP, CBD, FTDP-17 with N279K *MAPT* mutation) and 3R tauopathies (Pick’s disease, FTDP-17 with G272 *MAPT* mutation) compared to the weak signals of AV-1451. Additionally, PSP tau fibrils binding sites appeared to favor PBB3 over AV-1451.^71^

In addition to the off-target binding to neuromelanin reported in multiple studies, it has been reported that [^18^F]AV-1451 may bind to monoamine oxidase A (MAO-A).^72^ In vitro analysis demonstrated that a MAO-A ligand, clorgyline, is able to fully displace [^18^H]AV-1451.^72^ This off-target may interfere with tau imaging as MAO-A expression can be found in many brain regions.

### PET analysis

There are various methods to analyze PET data and determination of the appropriate approach for a given radiotracer is essential for accurate and reliable results. The following is a summary of kinetic validation studies performed with tau radiotracers.

A study performed with seven AD patients and seven healthy controls manually collected arterial samples while scanning with [^11^C]PBB3.^40^ BP_ND_ was estimated using dual input functions. The multilinear reference tissue model (MRTM) was used to estimate BP_ND_ using the cerebellar cortex as the reference region. SUVR-1 at 30–50 min was also calculated and both reference tissue method results correlated with MRTM BP_ND_, suggesting that these simplified methods are appropriate for quantifying [^11^C]PBB3 uptake.^40^

Two kinetic studies have been performed with [^18^F]AV-1451 to determine whether simplified methods of analysis are capable of effectively measuring radiotracer retention. One study involved four healthy controls, two subjects with MCI and three with a history of traumatic brain injury.^73^ Arterial samples were obtained and used to calculate time activity curves for whole blood and plasma. The two-tissue compartmental model demonstrated good fits and correlated with reference tissue methods using the cerebellar cortex: DVR and SUVR. SUVR from 80–100 min was deemed comparable to that of 100–120 min; therefore, 80–100 min might be the most appropriate time interval.^73^ Another kinetic study utilizing [^18^F]AV-1451 and arterial samples from four young healthy controls, four older healthy controls and eight subjects with AD reported consistent results. There was a strong correlation between SUVR from 80–100 min and distribution volume calculated based on the two tissue compartment model.^74^

## Conclusion

The recent development of PET radiotracers capable of imaging tau pathology has led to testing in parkinsonian tauopathies. Originally designed and tested in AD, these radiotracers must undergo full testing in non-AD tauopathies to validate their use in disorders such as PSP, CBD, and FTPD-17. While tracers such as [^18^F]FDDNP and [^11^C]PBB3 have demonstrated binding in PSP and CBD in preliminary studies, their efficacy may be limited by off-target binding to β-sheet structures and white matter, respectively. THK derivatives [^18^F]THK-5317 and [^18^F]THK-5351 have also demonstrated promising results in studies with PSP and CBD. However, replication of these studies with larger sample sizes, in addition to correlations with ante-mortem and post-mortem data are required to validate these radiotracers. Finally, [^18^F]AV-1451 has been used in numerous studies with PSP and CBD, with varying results. In PSP, [^18^F]AV-1451 has shown to bind brain regions with known tau pathology; however, there is overlap with healthy controls, which may decrease clinically utility and these results are not replicated in post-mortem data. Further testing in CBD with larger sample sizes and control groups is required to fully understand the efficacy of [^18^F]AV-1451 in CBD. The off-target binding of, [^18^F]AV-1451 to MAO-A should be further investigated, both in vitro and in vivo.

### Data availability

Data sharing not application to this article as no datasets were generated or analyzed during the current study.

## References

[1] Avila J, Lucas JJ, Perez M, Hernandez F (2004). Role of tau protein in both physiological and pathological conditions. Physiol. Rev..

[2] Spillantini MG, Goedert M (2013). Tau pathology and neurodegeneration. Lancet Neurol..

[3] De Silva R (2003). Pathological inclusion bodies in tauopathies contain distinct complements of tau with three or four microtubule-binding repeat domains as demonstrated by new specific monoclonal antibodies. Neuropathol. Appl. Neurobiol..

[4] Iqbal K, Liu F, Gong C-X, Grundke-Iqbal I (2010). Tau in Alzheimer disease and related tauopathies. Curr. Alzheimer Res..

[5] Shahani N, Brandt R (2002). Functions and malfunctions of the tau proteins. Cell. Mol. Life Sci..

[6] Noble W, Hanger DP, Miller CCJ, Lovestone S (2013). The importance of tau phosphorylation for neurodegenerative diseases. Front. Neurol..

[7] Golbe L (2014). The tau of PSP: a long road to treatment. Mov. Disord..

[8] Liscic RM, Srulijes K, Gröger A, Maetzler W, Berg D (2013). Differentiation of progressive supranuclear palsy: clinical, imaging and laboratory tools. Acta Neurol. Scand..

[9] Osaki Y (2004). Accuracy of clinical diagnosis of progressive supranuclear palsy. Mov. Disord..

[10] Golbe LI (2014). Progressive supranuclear palsy. Semin. Neurol..

[11] Hoglinger, G. et al. Clinical diagnosis of progressive supranuclear palsy–the movement disorder society criteria. *Mov. Disord.* **32**, 853–864 (2017). 10.1002/mds.26987 PMC5516529 28467028

[12] Kobylecki C (2015). Cognitive–behavioural features of progressive supranuclear palsy syndrome overlap with frontotemporal dementia. J. Neurol..

[13] Arai T (2001). Distinct isoforms of tau aggregated in neurons and glial cells in brains of patients with Pick’s disease, corticobasal degeneration and progressive supranuclear palsy. Acta Neuropathol..

[14] Zhukareva V (2006). Unexpected abundance of pathological tau in progressive supranuclear palsy white matter. Ann. Neurol..

[15] Dickson DW (1999). Neuropathologic differentiation of progressive supranuclear palsy and corticobasal degeneration. J. Neurol..

[16] Armstrong RA, Cairns NJ (2013). Spatial patterns of the tau pathology in progressive supranuclear palsy. Neurol. Sci..

[17] Tawanna K, Ramsden DB (2001). Progressive supranuclear palsy. J. Clin. Pathol. Mol. Pathol.

[18] Togo T, Dickson DW (2002). Tau accumulation in astrocytes in progressive supranuclear palsy is a degenerative rather than a reactive process. Acta. Neuropathol..

[19] Wray S, Saxton M, Anderton BH, Hanger DP (2008). Direct analysis of tau from PSP brain identifies new phosphorylation sites and a major fragment of N-terminally cleaved tau containing four microtubule-binding repeats. J. Neurochem..

[20] Grijalvo-Perez A, Litvan I (2014). Corticobasal degeneration. Semin. Neurol..

[21] Armstrong MJ (2013). Criteria for the diagnosis of corticobasal degeneration. Neurology.

[22] Ludolph AC (2009). Tauopathies with Parkinsonism: clinical spectrum, neuropathologic basis, biological markers, and treatment options. Eur. J. Neurol..

[23] Poewe W, Wenning G (2002). The differential diagnosis of Parkinson’s disease. Eur. J. Neurol..

[24] Stamelou M, Bhatia KP (2015). Atypical Parkinsonism: diagnosis and treatment. Neurol. Clin..

[25] Thal DR, Attems J, Ewers M (2014). Spreading of amyloid, tau, and microvascular pathology in Alzheimer’s disease: findings from neuropathological and neuroimaging studies. J. Alzheimer’s Dis.

[26] Takahashi M (2002). Morphological and biochemical correlations of abnormal tau filaments in progressive supranuclear palsy. J. Neuropathol. Exp. Neurol..

[27] Ahmed RM (2014). Biomarkers in dementia: clinical utility and new directions. J. Neurol. Neurosurg. Psychiatry.

[28] McMillan CT (2013). White matter imaging helps dissociate tau from TDP-43 in frontotemporal lobar degeneration. J. Neurol. Neurosurg. Psychiatry.

[29] Warren J, Rohrer J, Rossor M (2013). Frontotemporal dementia. Lancet Neurol..

[30] Spillantini MG, Bird TD, Ghetti B (1998). Frontotemporal dementia and Parkinsonism linked to chromosome 17: a new group of tauopathies. Brain Pathol..

[31] Espay AJ, Litvan I (2011). Parkinsonism and frontotemporal dementia: the clinical overlap. J. Mol. Neurosci..

[32] Pike VW (2010). PET Radiotracers crossing the blood-brain barrier and surviving metabolism. Trends Pharmacol. Sci..

[33] Villemagne VL, Okamura N (2014). In vivo tau imaging: obstacles and progress. Alzheimer’s Dement..

[34] Shah M, Catafau AM (2014). Molecular imaging insights into neurodegeneration: focus on tau PET radiotracers. J. Nucl. Med..

[35] Shoghi-Jadid K (2002). Localization of neurofibrillary tangles and beta-amyloid plaques in the brains of living patients with Alzheimer disease. Am. J. Geriatr. Psychiatry.

[36] Small GW (2006). PET of brain amyloid and tau in mild cognitive impairment. N. Engl. J. Med..

[37] Kepe V (2013). PET imaging of neuropathology in tauopathies: progressive supranuclear palsy. J. Alzheimers Dis..

[38] Maruyama M (2013). Article imaging of tau pathology in a tauopathy mouse model and in Alzheimer patients compared to normal controls. Neuron.

[39] Braak H, Braak E, Bohl J (1993). Staging of Alzheimer-related cortical destruction. Eur. Neurol..

[40] Kimura Y (2015). PET quantification of tau pathology in human brain with 11C-PBB3. J. Nucl. Med..

[41] Fodero-Tavoletti MT (2011). 18F-THK523: a novel in vivo tau imaging ligand for Alzheimer’s disease. Brain.

[42] Harada R (2013). Comparison of the binding characteristics of [18F]THK-523 and other amyloid imaging tracers to Alzheimer’s disease pathology. Eur. J. Nucl. Med. Mol. Imaging.

[43] Villemagne, V. L. et al. In vivo evaluation of a novel tau imaging tracer for Alzheimer’ s disease. 816–826. doi:10.1007/s00259-013-2681-7 (2014). 10.1007/s00259-013-2681-7 24514874

[44] Fodero-Tavoletti M, Furumoto S (2014). Assessing THK523 selectivity for tau deposits in Alzheimer’s disease and non–Alzheimer’s disease tauopathies. Alzheimers Res. Ther.

[45] Okamura N (2013). Novel 18F-labeled arylquinoline derivatives for noninvasive imaging of tau pathology in Alzheimer disease. J. Nucl. Med..

[46] Harada R (2015). [^18^F]THK-5117 PET for assessing neurofibrillary pathology in Alzheimer’s disease. Eur. J. Nucl. Med. Mol. Imaging.

[47] Ishiki A (2015). Longitudinal assessment of tau pathology in patients with Alzheimer’s disease using [18F]THK-5117 positron emission tomography. PLoS ONE.

[48] Saint-Aubert L (2016). Regional tau deposition measured by [18F]THK5317 positron emission tomography is associated to cognition via glucose metabolism in Alzheimer’s disease. Alzheimer’s Res. Ther.

[49] Chiotis K (2016). Imaging in-vivo tau pathology in Alzheimer’s disease with THK5317 PET in a multimodal paradigm. Eur. J. Nucl. Med. Mol. Imaging.

[50] Harada R (2015). 18F-THK5351: a novel PET radiotracer for imaging neurofibrillary pathology in Alzheimer’s disease. J. Nucl. Med..

[51] Ishiki A (2016). Tau imaging with [^18^F]THK-5351 in progressive supranuclear palsy. Eur. J. Neurol..

[52] Xia CF (2013). [(18)F]T807, a novel tau positron emission tomography imaging agent for Alzheimer’s disease. Alzheimers Dement..

[53] Marquie M (2015). Validating novel tau positron emission tomography tracer [F-18]-AV-1451 (T807) on postmortem brain tissue. Ann. Neurol..

[54] Sander K (2016). Characterization of tau positron emission tomography tracer [F] AV-1451 binding to postmortem tissue in Alzheimer’s disease, primary tauopathies, and other dementias. Alheimer’s Dement..

[55] Lowe VJ (2016). An autoradiographic evaluation of AV-1451 Tau PET in dementia. Acta Neuropathol. Commun.

[56] Chien DT (2013). Early clinical PET imaging results with the novel PHF-tau radioligand [F-18]-T807. J. Alzheimer’s Dis..

[57] Schwarz AJ (2016). Regional profiles of the candidate tau PET ligand 18F-AV-1451 recapitulate key features of Braak histopathological stages. Brain.

[58] Sepulcre J (2016). In vivo tau, amyloid, and gray matter profiles in the aging brain. J. Neurosci..

[59] Schöll M (2016). PET imaging of tau deposition in the aging human brain. Neuron.

[60] Cho H (2016). Tau PET in Alzheimer disease and mild cognitive impairment. Neurology.

[61] Ossenkoppele R (2016). Tau PET patterns mirror clinical and neuroanatomical variability in Alzheimer’s disease. Brain.

[62] Chhatwal JP (2016). Temporal T807 binding correlates with CSF tau and phospho-tau in normal elderly. Neurology.

[63] Smith R (2016). Increased basal ganglia binding of 18F-AV-1451 in patients with progressive supranuclear palsy. Mov. Disord..

[64] Whitwell JL (2016). [^18^F]AV-1451 tau positron emission tomography in progressive supranuclear palsy. Mov. Disord..

[65] Cho H (2016). Subcortical ^18^F-AV-1451 binding patterns in progressive supranuclear palsy. Mov. Disord..

[66] Coakeley, S. et al. Positron emission tomography imaging of tau pathology in progressive supranuclear palsy. *J. Cereb. Blood. Flow Metab*. doi:10.1177/0271678X16683695 (2017). 10.1177/0271678X16683695 PMC5584690 28155586

[67] Passamonti, L. et al. 18F-AV-1451 positron emission tomography in Alzheimer’s disease and progressive supranuclear palsy. *Cereb. Cortex*. doi:10.1093/cercor/bhw393 (2017). 10.1093/brain/aww340 PMC5382948 28122879

[68] Marquié M (2017). Pathologic correlations of [F-18]-AV-1451 imaging in non-Alzheimer tauopathies. Ann. Neurol..

[69] Josephs KA (2016). [18F]AV-1451 tau-PET uptake does correlate with quantitatively measured 4R-tau burden in autopsy-confirmed corticobasal degeneration. Acta Neuropathol..

[70] McMillan CT (2016). Multimodal evaluation demonstrates in vivo 18F-AV-1451 uptake in autopsy-confirmed corticobasal degeneration. Acta Neuropathol..

[71] Ono M (2017). Distinct binding of PET ligands PBB3 and AV-1451 to tau fibril strains in neurodegenerative tauopathies. Brain.

[72] Vermeiren C (2015). T807, a reported selective tau tracer, binds with nanomolar affinity to monoamine oxidase a. Alzheimer’s Dement..

[73] Wooten D (2016). Pharmacokinetic evaluation of the tau PET radiotracer [18F]T807 ([18F]AV-1451) in human subjects. J. Nucl. Med..

[74] Barret O (2016). Kinetic modeling of the tau PET tracer 18F-AV-1451 in human healthy volunteers and Alzheimer’s disease subjects. J. Nucl. Med..

